# Prognostic Value of a Novel Risk Score Combining Psoas Muscle Density and ALBI Grade in Localized Renal Cell Carcinoma

**DOI:** 10.3390/medsci14050509

**Published:** 2026-08-24

**Authors:** Tomoyuki Makino, Kouji Izumi, Ryunosuke Nakagawa, Taiki Kamijima, Suguru Kadomoto, Renato Naito, Hiroaki Iwamoto, Hiroshi Yaegashi, Kazuyoshi Shigehara, Takahiro Nohara, Atsushi Mizokami

**Affiliations:** Department of Integrative Cancer Therapy and Urology, Kanazawa University Graduate School of Medical Science, 13-1 Takara-Machi, Kanazawa 920-8641, Japan; azuizu2003@yahoo.co.jp (K.I.); r_a_rhero0226southern@yahoo.co.jp (R.N.); kamiji0029@yahoo.co.jp (T.K.); soogle58@gmail.com (S.K.); thealfuu@yahoo.co.jp (R.N.); hiroaki017@yahoo.co.jp (H.I.); hyae2002jp@yahoo.co.jp (H.Y.); kshigehara0415@yahoo.co.jp (K.S.); t_nohara704@yahoo.co.jp (T.N.); mizokami@staff.kanazawa-u.ac.jp (A.M.)

**Keywords:** renal cell carcinoma, psoas muscle density, ALBI grade, myosteatosis, cancer cachexia, prognosis

## Abstract

**Background:** Sarcopenia, systemic inflammation, and malnutrition are established poor prognostic factors in renal cell carcinoma (RCC). This study investigated the utility of a novel preoperative score combining psoas muscle density (PMD)—an imaging-based indicator of muscle quality—and the albumin–bilirubin (ALBI) grade—a blood-based biomarker of liver reserve and systemic nutritional status—for predicting disease-free survival (DFS) and overall survival (OS) in patients undergoing curative surgery for RCC. **Methods:** This retrospective observational study included 274 patients with non-metastatic RCC treated with radical or partial nephrectomy. Preoperative computed tomography was utilized to measure PMD, defining “low PMD” as a value below the sex-specific median (males: 47.75 Hounsfield Units [HU]; females: 46.25 HU). “Worsened ALBI” was defined as an ALBI grade ≥ 2. Patients were stratified into three risk categories: Score 0 (both normal, *n* = 122), Score 1 (either abnormal, *n* = 114), and Score 2 (both abnormal, *n* = 38). **Results:** Kaplan–Meier analysis revealed a highly significant, stepwise decline in both DFS and OS as the risk score increased (log–rank *p* < 0.001 and *p* = 0.004, respectively). Multivariate Cox regression identified the combined risk score as a robust, independent prognostic factor for DFS (Score 1: HR 1.93, 95% CI 1.11–3.37, *p* = 0.020; Score 2: HR 3.58, 95% CI 1.82–7.02, *p* < 0.001). Furthermore, after adjusting for age and comorbidities, the score remained an independent predictor of poor OS (Score 1: HR 2.35, 95% CI 1.08–5.13, *p* = 0.032; Score 2: HR 3.01, 95% CI 1.16–7.82, *p* = 0.024). The combined model synergistically enhanced risk stratification accuracy compared to evaluating either factor independently. **Conclusions:** The concurrent presence of preoperative low PMD and a worsened ALBI grade is a powerful, independent predictor of poor prognosis in localized RCC. Derived solely from routine preoperative imaging and laboratory tests, this straightforward scoring system effectively captures the structural and immunometabolic dimensions of cancer cachexia and host vulnerability. This tool can significantly aid in personalizing postoperative surveillance strategies and identifying high-risk patients who may warrant closer postoperative surveillance or who might be considered as high-risk candidates for adjuvant therapy discussions.

## 1. Introduction

Renal cell carcinoma (RCC) is one of the most common urological malignancies worldwide, with a rising incidence driven by the widespread use of cross-sectional abdominal imaging [1,2]. Although surgical management via radical or partial nephrectomy remains the cornerstone of treatment with curative intent [3], a significant proportion of patients with localized disease eventually experience tumor recurrence, leading to a subsequent decline in overall survival (OS). While traditional anatomical staging (TNM classification) [4] and histological grading (ISUP/Fuhrman grade) [5] remain the gold standards for risk assessment, their predominantly tumor-centric focus overlooks critical host-related factors. Consequently, there is an unmet clinical need for objective biomarkers that accurately reflect the host’s physiological status, vulnerability, and systemic reserve to optimize postoperative management.

Accumulating evidence indicates that host-related factors—particularly skeletal muscle depletion (sarcopenia), systemic inflammation, and malnutrition—play a pivotal role in the pathogenesis and progression of various malignancies [6,7,8,9]. While the psoas muscle index has traditionally been used to define sarcopenic muscle mass [10,11], recent attention has shifted toward psoas muscle density (PMD) as a superior primary indicator of muscle quality [12,13,14]. Low muscle attenuation, indicative of myosteatosis, serves as an objective radiological proxy for physical frailty and advanced cancer cachexia [15]. However, the prognostic power of PMD alone is often moderate, as its clinical impact can be heavily confounded by advancing age, severe comorbidities, or aggressive tumor characteristics.

Concurrently, the albumin–bilirubin (ALBI) grade—originally established for assessing liver reserve in hepatocellular carcinoma [16,17,18]—has emerged as a powerful, objective biomarker of systemic nutritional and inflammatory status in non-hepatic malignancies [19,20,21]. A worsened ALBI grade reflects systemic host depletion and immunometabolic dysfunction across several solid tumors, including urological cancers [22].

Given that structural muscle wasting and systemic inflammation are interconnected, synergistic facets of cancer cachexia [23,24], we hypothesized that integrating muscle quality and systemic reserve would provide a more holistic and accurate representation of host vulnerability. Therefore, this study aimed to develop a novel, highly practical preoperative risk score combining PMD and the ALBI grade in patients undergoing curative resection for localized RCC, evaluating its efficacy in stratifying post-surgical disease-free survival (DFS) and OS.

## 2. Materials and Methods

### 2.1. Patient Selection and Study Design

This retrospective observational study was approved by the institutional review board, and the requirement for informed consent was waived. A query of our institutional database was performed to identify 432 patients who underwent interventions for renal tumors and had available psoas muscle measurements between 2007 and 2018. To establish a homogeneous cohort of patients with localized disease treated with curative intent, we excluded those with distant metastasis or unknown metastatic status (*n* = 86) and those who received non-surgical treatments or non-curative resections (*n* = 40). Patients with missing data for core variables required for multivariate analysis were also excluded (*n* = 32). Consequently, the final analytical cohort comprised 274 patients with non-metastatic (M0) RCC who underwent radical or partial nephrectomy. Although RCC encompasses a heterogeneous group of histological subtypes, all non-metastatic patients were initially included to evaluate the score’s broad prognostic applicability. To specifically address the potential confounding effect of histological heterogeneity, a dedicated subgroup analysis restricting the cohort to clear cell RCC was conducted.

### 2.2. Evaluation of Psoas Muscle Density (PMD)

Skeletal muscle parameters were evaluated using preoperative unenhanced computed tomography (CT) scans performed within three months prior to surgery. Cross-sectional areas and attenuation values of the bilateral psoas muscles were measured at the level of the third lumbar vertebra. PMD was calculated as the mean radiation attenuation value in Hounsfield Units (HU). We adopted sex-specific medians (males: 47.75 HU; females: 46.25 HU) as cut-offs to define muscle quality, classifying values below these thresholds as “Low PMD”.

Of note, while an exploratory receiver operating characteristic (ROC) curve analysis targeting 5-year recurrence yielded an optimal cut-off of 47.95 HU for males—demonstrating near-perfect agreement with the cohort median—the corresponding model for the female subgroup lacked sufficient predictive power (AUC = 0.369). This limitation was primarily driven by the sparse number of recurrence events among female patients, which precluded reliable mathematical stratification. Consequently, maintaining the predefined median thresholds was determined to be the most robust and clinically pragmatic approach for all subsequent risk stratification models.

### 2.3. Assessment of ALBI Grade

Preoperative laboratory data obtained within 30 days prior to surgery were used to calculate the ALBI score using the validated formula: ALBI score = (log_10_ total bilirubin in μmol/L × 0.66) + (serum albumin in g/L × −0.085). For standard clinical laboratory values provided in mg/dL, total bilirubin was converted to μmol/L (1 mg/dL = 17.1 μmol/L). The conventional ALBI grade thresholds were applied, where Grade 1 is defined as a score ≤ −2.60, Grade 2 as >−2.60 to ≤−1.39, and Grade 3 as >−1.39. To maximize diagnostic sensitivity for systemic host vulnerability, “Worsened ALBI” was defined as an ALBI grade ≥ 2, representing sub-optimal hepatic reserve, ongoing systemic inflammation, or mild-to-moderate malnutrition.

### 2.4. Development of the Combined Risk Score

The novel preoperative risk score was constructed by assigning 1 point for each abnormal host parameter: 1 point for Low PMD (below the sex-specific median) and 1 point for Worsened ALBI (ALBI grade ≥ 2). Based on the total accumulated points, patients were stratified into three distinct risk categories: Score 0 (0 points: normal PMD and ALBI grade 1, *n* = 122), Score 1 (1 point: either low PMD or ALBI grade ≥ 2, *n* = 114), and Score 2 (2 points: coexistence of low PMD and ALBI grade ≥ 2, *n* = 38).

### 2.5. Statistical Analysis

The primary endpoints of this study were disease-free survival (DFS), defined as the time from surgery to the documentation of tumor recurrence, distant metastasis, or death from any cause, and overall survival (OS), defined as the time from surgery to death from any cause. Survival curves were generated using the Kaplan–Meier method, and differences between the risk score groups were assessed using the log-rank test. The 5-year survival probabilities were calculated for each respective group.

To determine independent predictors of DFS and OS, univariate and multivariate Cox proportional hazards regression analyses were performed. Variables with established clinical relevance that demonstrated statistical significance (*p* < 0.05) in the initial univariate analyses were included in the respective multivariate models. To minimize residual confounding, the final multivariate model for DFS was adjusted for aggressive tumor characteristics (pT stage ≥ 3, Fuhrman grade ≥ 3, and positive LVI), whereas the model for OS was adjusted for critical host background features (age ≥ 63 years and Charlson Comorbidity Index [CCI] ≥ 3). Hazard ratios (HRs) and 95% confidence intervals (CIs) were computed. All statistical analyses were conducted using GraphPad Prism (version 6.07; GraphPad Software Inc., San Diego, CA, USA) and Statistical Package for the Social Sciences (version 31.0; IBM Corp., Armonk, NY, USA). All tests were two-sided, and a *p*-value < 0.05 was considered statistically significant.

## 3. Results

### 3.1. Patient Characteristics

The clinicopathological characteristics of the 274 patients included in the final analysis are comprehensively presented in Table 1. The median observation periods were 7.1 years (interquartile range [IQR]: 4.9–10.5). The median age of the overall cohort was 63.0 years (IQR: 55.0–71.0), and 199 patients (72.6%) were male. When stratified by the proposed preoperative risk score, the median age was significantly lower in the Score 0 group (57.0 years) compared to the Score 1 (67.0 years) and Score 2 (66.5 years) groups. Higher risk scores were associated with advanced comorbidities (CCI ≥ 3), larger tumor sizes, and higher rates of advanced pathological features, including pT stage ≥ 3 and Fuhrman grade ≥ 3. During the follow-up period, recurrence events occurred in 15 patients (12.3%), 15 patients (13.2%), and 13 patients (34.2%) in the Score 0, 1, and 2 groups, respectively.

### 3.2. Prognostic Value of the Combined Risk Score for DFS

**Figure 1:**
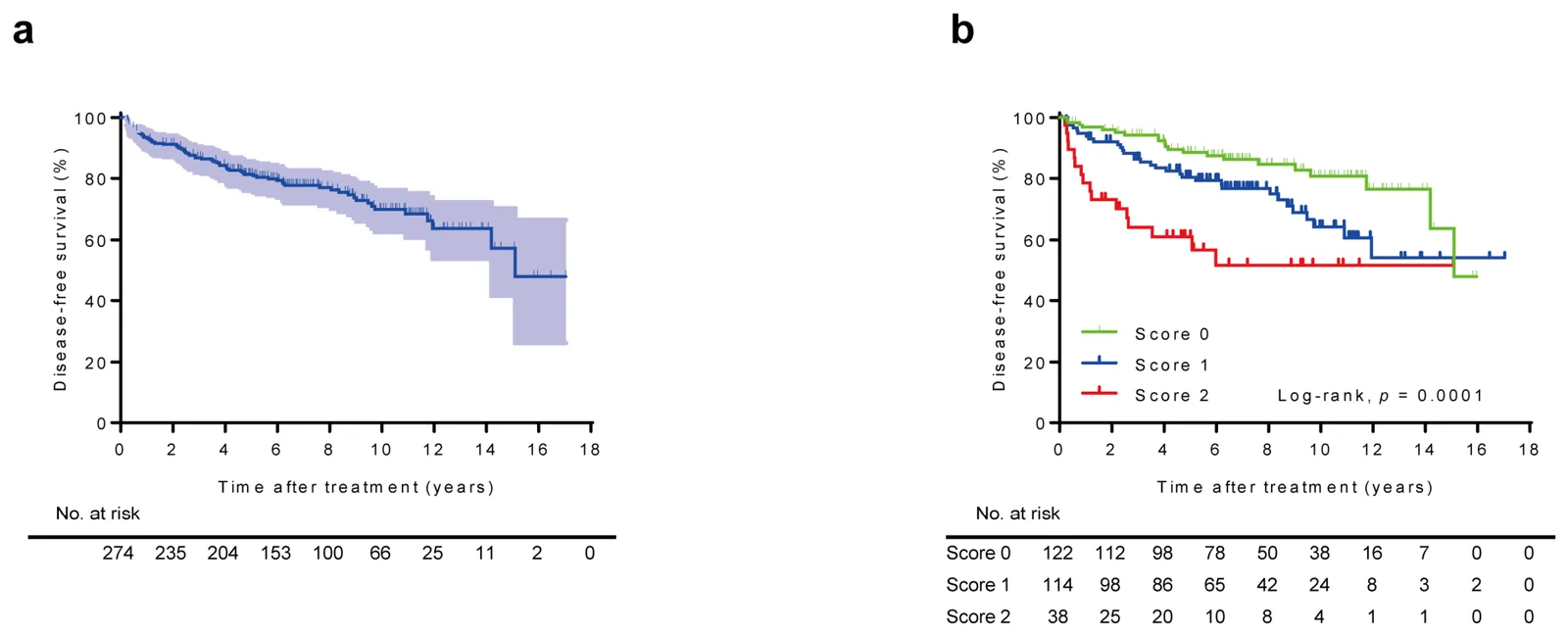
Kaplan–Meier curves for Disease-Free Survival (DFS) (*n* = 274). (**a**) DFS curve for the entire patient cohort and (**b**) DFS curves stratified according to the Risk Score tiers.

In the entire patient cohort, the DFS curve depicted a generally favorable prognosis typical of localized disease following curative resection (Figure 1a). When stratified by the risk model, however, Kaplan–Meier analysis demonstrated that higher preoperative risk scores were strongly associated with a stepwise and statistically significant decline in DFS (Figure 1b; log–rank, *p* < 0.001). As illustrated in Figure 1b, the survival curve for the Score 2 group exhibited a visibly steeper decline, which is also reflected by a rapid reduction in the corresponding number of patients at risk over the observation period. The 5-year DFS rates were 88.4%, 80.4%, and 61.0% for Scores 0, 1, and 2, respectively, revealing a substantial increase in early recurrence or crossover mortality among patients burdened with concurrent host deficits.

In the univariate Cox proportional hazards analysis (Table 2), advanced tumor stage (pT ≥ 3: HR 4.11, *p* < 0.001), high Fuhrman grade (Grade ≥ 3: HR 4.09, *p* < 0.001), and positive LVI (HR 2.42, *p* < 0.001) were significant predictors of poor DFS. The combined risk score also demonstrated highly robust predictive power in the univariate analysis; Score 1 carried an HR of 1.84 (95% CI: 1.06–3.19, *p* = 0.031) and Score 2 carried an HR of 3.91 (95% CI: 2.02–7.54, *p* < 0.001) relative to the Score 0 reference group.

Crucially, in the multivariate Cox regression model adjusting for all relevant clinical and pathological covariates, the combined risk score maintained its status as a robust and independent predictor of DFS. Compared with the reference group (Score 0), the adjusted HR was 1.93 (95% CI: 1.11–3.37, *p* = 0.020) for Score 1, and drastically increased to 3.58 (95% CI: 1.82–7.02, *p* < 0.001) for Score 2. Among other traditional factors, only pT stage ≥ 3 (HR 2.67, *p* < 0.001) and Fuhrman grade ≥ 3 (HR 2.75, *p* < 0.001) remained independent predictors, whereas LVI lost its statistical significance in the multivariate setting (HR 1.48, *p* = 0.197).

### 3.3. Prognostic Value of the Combined Risk Score for OS

**Figure 2:**
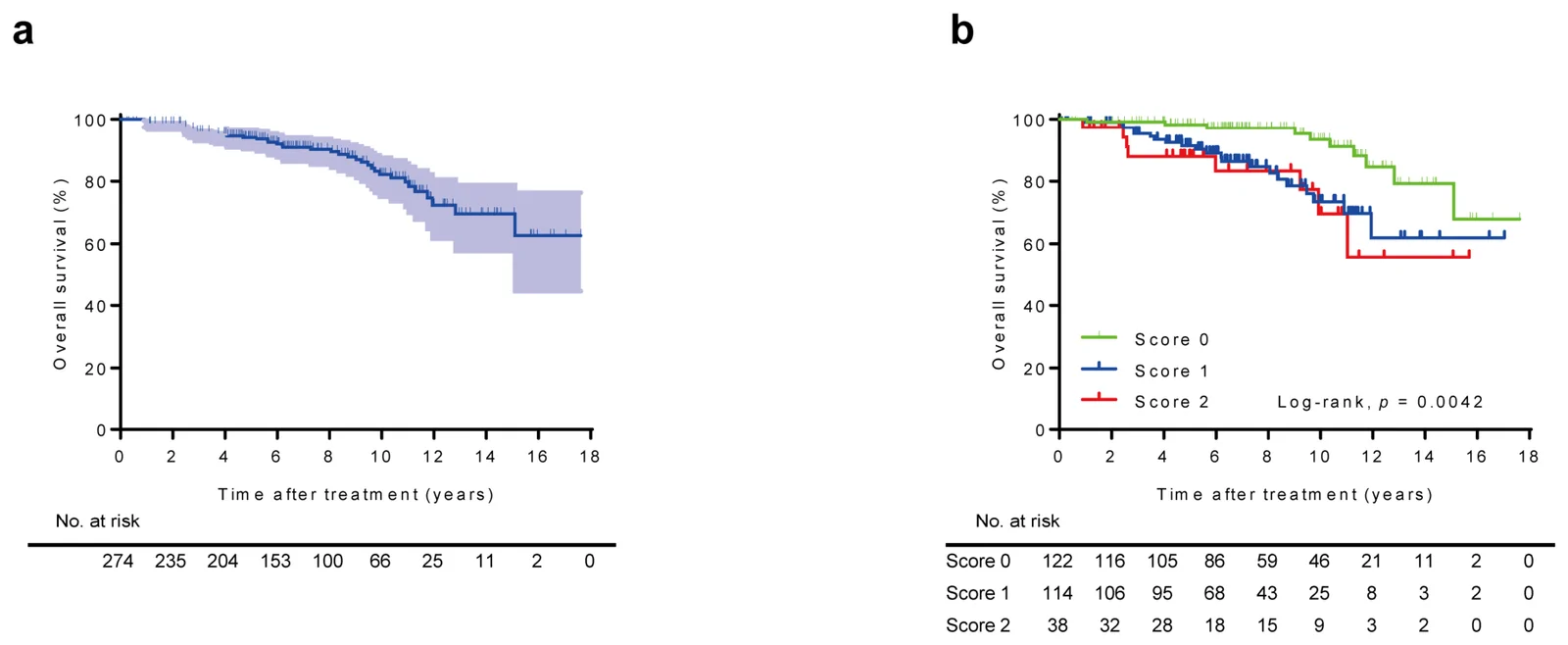
Kaplan–Meier curves for Overall Survival (OS) (*n* = 274). (**a**) OS curve for the entire patient cohort, and (**b**) OS curves stratified according to the Risk Score tiers.

Similarly, while the long-term OS remained exceedingly high for the overall patient cohort (Figure 2a), the preoperative risk score successfully stratified long-term survival outcomes for OS. Kaplan–Meier survival curves indicated a clear, significant separation among the three risk tiers (Figure 2b; log–rank, *p* = 0.004). Consistent with the DFS findings, the visual separation of the curves in Figure 2b further illustrates the persistently lower OS probability for patients in the Score 2 category, corresponding to the lowest number of surviving patients at risk over time. The 5-year OS rates were 98.2%, 91.4%, and 87.9% for Scores 0, 1, and 2, respectively.

Univariate Cox analysis for OS (Table 3) identified age ≥ 63 years (HR 3.40, *p* < 0.001), CCI ≥ 3 (HR 2.58, *p* = 0.004), and the combined risk score as significant risk factors for death. Compared to Score 0, Score 1 carried a univariate HR of 2.97 (95% CI: 1.39–6.34, *p* = 0.005) and Score 2 carried a univariate HR of 3.64 (95% CI: 1.43–9.26, *p* = 0.007). In contrast, standard tumor characteristics like pT stage (*p* = 0.137), Fuhrman grade (*p* = 0.535), and LVI (*p* = 0.238) did not reach statistical significance for OS in this specifically localized cohort.

In the multivariate Cox regression model for OS, after adjusting for age and comorbidities, the combined risk score remained an independent and strong predictor of all-cause mortality. Patients in the Score 1 group exhibited an adjusted HR of 2.35 (95% CI: 1.08–5.13, *p* = 0.032), while those in the Score 2 group exhibited an adjusted HR of 3.01 (95% CI: 1.16–7.82, *p* = 0.024) relative to the Score 0 group. Notably, age and CCI lost their independent statistical significance within the fully adjusted model, further highlighting the overarching and robust prognostic power of the integrated host score.

### 3.4. Individual Parameter Analysis

To evaluate the prognostic value of PMD and ALBI grade independently, we conducted separate sub-analyses (Supplementary Table S1). For DFS, low PMD alone did not demonstrate a significant association in univariate analysis, though it emerged as a significant independent risk factor when adjusted for pT stage, Grade, and LVI. ALBI grade ≥ 2 alone was a strong standalone predictor in both univariate and multivariate adjusted analyses for DFS. For OS, the prognostic value of PMD alone was significant in univariate analysis but lost independent significance upon adjustment for age and CCI. Conversely, ALBI risk alone remained highly significant for OS across all analytical settings, underscoring the necessity of their integration for maximal predictive accuracy.

### 3.5. Subgroup Analysis in Clear Cell RCC (ccRCC)

Given the biological heterogeneity of RCC, we performed a dedicated subgroup analysis restricted to patients with the clear cell histology (ccRCC, *n* = 244) to minimize potential histological confounding. Consistent with the overall cohort findings, the combined risk score maintained robust prognostic value in the ccRCC subgroup. In the multivariate Cox regression analysis adjusting for aggressive tumor factors (pT stage, Fuhrman grade, and LVI), Score 2 remained a strong, independent predictor of poor DFS (HR 3.91, 95% CI 1.91–8.00, *p* < 0.001). Similarly, after adjusting for patient background characteristics (age and CCI), the score remained an independent predictor of poor OS (Score 1: HR 2.72, 95% CI 1.11–6.67, *p* = 0.029; Score 2: HR 3.74, 95% CI 1.30–10.71, *p* = 0.014). The detailed results of this ccRCC subgroup analysis are presented in Supplementary Table S2.

## 4. Discussion

In the present study, we successfully developed and validated a novel, highly practical preoperative risk score by combining PMD and the ALBI grade in patients with non-metastatic RCC undergoing radical or partial nephrectomy. Our findings unequivocally demonstrate that an increase in this composite score is an independent and robust predictor of both post-surgical recurrence and long-term all-cause mortality. More importantly, our data revealed a profound synergistic effect: while PMD alone offered limited or borderline independent predictive power due to the confounding nature of clinical variables, its integration with the ALBI grade allowed for the precise identification of an ultra-high-risk patient population (Score 2). This population is characterized by a 3.58-fold increased risk of recurrence and a 3.01-fold increased risk of death compared to patients with normal host parameters.

The biological mechanisms underlying the prognostic value of this combined score can be explained by the complex, interrelated pathways of cancer cachexia, systemic inflammation, and immunological host depletion [23,24]. Low PMD is an established radiological proxy for myosteatosis, characterized by the accumulation of microscopic fat droplets within skeletal muscle fibers [12,15]. Unlike a simple loss of muscle mass (sarcopenia quantity) [11,25], myosteatosis reflects qualitative muscle degeneration driven by chronic low-grade inflammation, localized oxidative stress, and profound mitochondrial dysfunction [13,14]. In the context of malignancy, tumor-derived pro-inflammatory cytokines—such as tumor necrosis factor-alpha (TNF-α), interleukin-6 (IL-6), and interferon-gamma (INF-γ)—play a central role in driving a systemic, simultaneous catabolic response across multiple organs. Specifically, these circulating cytokines not only alter lipid metabolism to promote rapid skeletal muscle proteolysis and subsequent myosteatosis, but they concurrently act on the liver to potently suppress albumin synthesis and induce an acute-phase response [24]. This shared, cytokine-driven biological crosstalk elegantly explains the synergistic prognostic value observed in our study: the combination of low PMD and a worsened ALBI grade captures both the structural and biochemical dimensions of this systemic inflammatory exhaustion. Consequently, low PMD represents a profound state of underlying physiological frailty and host vulnerability, which inherently impairs the patient’s capacity to withstand surgical stress and maintain active metabolic homeostasis, ultimately facilitating an environment permissive to micro-metastatic progression.

Concurrently, the ALBI grade serves as a highly sensitive, blood-based reflection of the host’s nutritional and inflammatory state. Serum albumin is not only a marker of nutritional intake and hepatic synthesis but is also a critical negative acute-phase reactant that is markedly suppressed during systemic inflammatory responses. Hypoalbuminemia actively impairs host immune surveillance, reduces antioxidant capacity, and compromises tissue repair mechanisms [26,27]. Total bilirubin, conversely, is a metabolic byproduct of heme catabolism that possesses potent endogenous antioxidant and anti-inflammatory properties within physiological ranges. Fluctuations in bilirubin levels intricately reflect underlying oxidative stress and systemic metabolic strain. Thus, an elevated ALBI grade integrates these two vital serum parameters into an objective, continuous index that captures immunometabolic exhaustion and host depletion far more accurately than traditional subjective nutritional tools.

The primary clinical and statistical significance of our study lies directly in the synergistic integration of these two distinct host-related dimensions. Cancer cachexia is a multi-organ syndrome involving systemic cross-talk between the primary tumor, skeletal muscle, and the liver [23]. As demonstrated in our separate sub-analysis, while the ALBI grade is a potent standalone marker, the prognostic value of PMD alone is easily obscured by advanced age or severe comorbidities. By merging the two into the integrated risk score, the subtle physiological effects of muscle quality degradation—reflecting hidden aging and frailty pathways—are successfully captured and superimposed onto the strong predictive foundation of the ALBI grade. Therefore, when a patient presents with both low PMD and a worsened ALBI grade (Score 2), it strongly indicates an advanced breakdown of systemic homeostasis. This signals an ultra-high-risk state that might be easily misclassified or underestimated by individual clinical evaluations. Crucially, our multivariate analyses confirmed that the PMD-ALBI score provides incremental prognostic information that extends beyond conventional clinical and pathological parameters. Even after directly adjusting for aggressive tumor characteristics—such as pT stage and Fuhrman grade—the integrated score remained a robust, independent predictor of both recurrence and mortality. This holistic approach definitively shifts the prognostic paradigm from purely tumor-focused staging toward a comprehensive assessment that balances aggressive tumor biology with host physiological resilience.

From a practical standpoint, this novel risk score offers exceptional clinical utility. It is derived entirely from routine, mandatory preoperative workups—specifically standard abdominal CT imaging and routine pre-surgical blood panels—requiring absolutely no additional financial cost, specialized equipment, or invasive testing. In the current era of expanding indications for postoperative adjuvant systemic therapies in RCC (such as immune checkpoint inhibitors) [28], identifying the ideal candidates who will derive the most benefit remains a major oncological challenge. Our score could serve as an invaluable clinical decision-making tool; patients classified as Score 2 may represent a distinct high-risk subpopulation requiring a highly rigorous, personalized postoperative imaging schedule. Furthermore, while prognostic tools do not directly predict treatment efficacy, identifying this ultra-high-risk subset can inform multidisciplinary discussions regarding the consideration of adjuvant interventions. Conversely, Score 0 patients exhibit excellent long-term survival and low recurrence rates, potentially allowing for more relaxed surveillance protocols to minimize radiation exposure, alleviate patient anxiety, and reduce healthcare costs.

Several limitations of the current study must be openly acknowledged. First, this was a retrospective observational study conducted at a single institution, which inherently introduces selection bias and limits the immediate broad generalizability of the findings. Second, while the overall cohort size (*n* = 274) was sufficient for robust multivariate modeling, the absolute number of recurrence and mortality events within certain subgroups—particularly among female patients—was small. This sparse event rate precluded the reliable mathematical derivation of optimal sex-specific ROC thresholds, necessitating the use of cohort medians. This reliance may affect the current reliability and clinical universality of the score specifically for women. Third, the evaluation was strictly limited to baseline preoperative metrics. We lacked sequential postoperative measurements for PMD and ALBI grade, leaving it unknown whether functional recovery or dynamic changes in these host markers correlate with altered survival trajectories. Fourth, regarding the timing of preoperative CT scans, we utilized a standard 3-month window; however, given that cancer cachexia can progress rapidly, future studies should investigate whether a narrower time window (e.g., within 1 month) yields even stronger prognostic accuracy. Fifth, as observed in the Kaplan–Meier survival curves, the number of patients at risk in the Score 2 group rapidly declined over the extended follow-up period. This reduction in the sample size at later time points widens the confidence intervals, meaning that the long-term survival estimates for this ultra-high-risk tier should be interpreted with appropriate statistical caution. Finally, to confirm whether these proposed PMD and ALBI cut-offs hold up across diverse patient demographics, external validation using independent, large-scale cohorts from multi-center international databases is imperative.

## 5. Conclusions

The preoperative combined risk score integrating psoas muscle density and ALBI grade represents a powerful, independent, and clinically practical prognostic model for patients with localized RCC undergoing curative resection. By successfully capturing the joint structural and immunometabolic dimensions of host vulnerability, this simple scoring system identifies an ultra-high-risk patient population prone to early recurrence and poor long-term survival. Given its complete reliance on standard routine clinical evaluations, this hybrid score can be effortlessly integrated into routine urological practice, facilitating personalized risk stratification and optimizing follow-up surveillance protocols. Before broad implementation into clinical practice, future validations using large-scale, multicenter cohorts are essential to confirm its universal applicability and to determine its potential role in guiding multidisciplinary discussions for high-risk patients.

## Figures and Tables

**Table 1:** Patient Characteristics According to the Combined Risk Score.

Characteristics	Overall (*N* = 274)	Score 0 (*N* = 122)	Score 1 (*N* = 114)	Score 2 (*N* = 38)
Age, years, median (IQR)	63.0 (55.0–71.0)	57.0 (47.0–65.0)	67.0 (60.0–74.0)	66.5 (62.0–76.0)
Male sex, *n* (%)	199 (72.6)	88 (72.1)	88 (77.2)	23 (60.5)
BMI, kg/m^2^, median (IQR)	23.7 (21.7–26.3)	23.2 (21.4–25.9)	24.0 (22.4–26.8)	23.6 (21.6–26.0)
CCI, median (IQR)	2.0 (1.0–3.0)	2.0 (1.0–3.0)	3.0 (2.0–3.0)	3.0 (2.0–3.8)
Tumor Size, mm, median (IQR)	28.5 (20.0–48.0)	26.0 (18.0–41.5)	30.0 (21.0–52.3)	33.5 (22.5–87.5)
pT stage ≥ 3, *n* (%)	46 (16.8)	15 (12.3)	20 (17.5)	11 (28.9)
Fuhrman grade ≥ 3, *n* (%)	49 (17.9)	14 (11.5)	20 (17.5)	15 (39.5)
LVI, positive, *n* (%)	131 (47.8)	56 (45.9)	57 (50.0)	18 (47.4)
Recurrence events, *n* (%)	43 (15.7)	15 (12.3)	15 (13.2)	13 (34.2)

BMI, body mass index; CCI, Charlson Comorbidity Index; IQR, interquartile range; LVI, lymphovascular invasion. Risk Score: Score 0 (Normal psoas muscle density [PMD] and ALBI grade 1), Score 1 (Low PMD or ALBI grade ≥ 2), Score 2 (Low PMD and ALBI grade ≥ 2).

**Table 2:** Univariate and Multivariate Cox Proportional Hazards Regression Analysis for Disease-Free Survival.

Variables	Categories	Univariate HR (95% CI)	*p*-Value	Multivariate HR (95% CI)	*p*-Value
Age, years	≥63 vs. <63	1.17 (0.73–1.89)	0.515	—	—
Sex	Male vs. Female	1.52 (0.84–2.73)	0.166	—	—
BMI, kg/m^2^	≥25 vs. <25	1.13 (0.69–1.86)	0.630	—	—
CCI	≥3 vs. <3	1.35 (0.84–2.18)	0.212	—	—
Histological type	Non-clear cell vs. Clear cell	0.76 (0.33–1.77)	0.529	—	—
pT stage	≥3 vs. <3	4.11 (2.51–6.73)	<0.001	2.67 (1.49–4.78)	<0.001
Fuhrman grade	≥3 vs. <3	4.09 (2.49–6.72)	<0.001	2.75 (1.62–4.68)	<0.001
LVI	Positive vs. Negative	2.42 (1.47–3.96)	<0.001	1.48 (0.82–2.66)	0.197
Risk Score	0	Reference	—	Reference	—
	1	1.84 (1.06–3.19)	0.031	1.93 (1.11–3.37)	0.020
	2	3.91 (2.02–7.54)	<0.001	3.58 (1.82–7.02)	<0.001

BMI, body mass index; CCI, Charlson Comorbidity Index; CI, confidence interval; HR, hazard ratio; LVI, lymphovascular invasion.

**Table 3:** Univariate and Multivariate Cox Proportional Hazards Regression Analysis for Overall Survival.

Variables	Categories	Univariate HR (95% CI)	*p*-Value	Multivariate HR (95% CI)	*p*-Value
Age, years	≥63 vs. <63	3.40 (1.64–7.03)	<0.001	2.09 (0.86–5.05)	0.102
Sex	Male vs. Female	1.69 (0.74–3.83)	0.211	—	—
BMI, kg/m^2^	≥25 vs. <25	0.99 (0.50–1.95)	0.966	—	—
CCI	≥3 vs. <3	2.58 (1.36–4.90)	0.004	1.58 (0.73–3.38)	0.243
Histological type	Non-clear cell vs. Clear cell	1.15 (0.45–2.95)	0.769	—	—
pT stage	≥3 vs. <3	1.73 (0.84–3.55)	0.137	—	—
Fuhrman grade	≥3 vs. <3	1.28 (0.59–2.79)	0.535	—	—
LVI	Positive vs. Negative	1.46 (0.78–2.76)	0.238	—	—
Risk Score	0	Reference	—	Reference	—
	1	2.97 (1.39–6.34)	0.005	2.35 (1.08–5.13)	0.032
	2	3.64 (1.43–9.26)	0.007	3.01 (1.16–7.82)	0.024

BMI, body mass index; CCI, Charlson Comorbidity Index; CI, confidence interval; HR, hazard ratio; LVI, lymphovascular invasion.

## Data Availability

The original contributions presented in this study are included in the article/Supplementary Material. Further inquiries can be directed to the corresponding author(s).

## Supplementary Materials

The following supporting information can be downloaded at https://www.mdpi.com/article/10.3390/medsci14050509/s1: Table S1. Univariate and Multivariate Analyses of Individual Parameters (PMD and ALBI Grade) for Disease-Free Survival (DFS) and Overall Survival (OS); Table S2. Multivariate Cox Proportional Hazards Regression Analysis for DFS and OS in the Clear Cell RCC Subgroup (*n* = 244).

## Abbreviations

The following abbreviations are used in this manuscript:

ALBI	Albumin–bilirubin
CCI	Charlson Comorbidity Index
CI	confidence interval
CT	computed tomography
DFS	disease-free survival
HR	hazard ratio
HU	Hounsfield Unit
IQR	interquartile range
LVI	lymphovascular invasion
OS	overall survival
PMD	psoas muscle density
RCC	renal cell carcinoma
ROC	receiver operating characteristic
