# Supplementary material for: Prognostic Value of a Novel Risk Score Combining Psoas Muscle Density and ALBI Grade in Localized Renal Cell Carcinoma
Source: Med Sci (Basel). 2026 Aug 24;14(5):509. doi: 10.3390/medsci14050509 (PMC13609120; doi:10.3390/medsci14050509)
Supplement: Supplementary file 1 [file medsci-14-00509-s001.zip › medsci-4498414-supplementary.pdf]

Prognostic Value of a Novel Risk Score Combining Psoas Muscle Density and ALBI Grade in Localized Renal Cell Carcinoma

Table S1. Univariate and Multivariate Analyses of Individual Parameters (PMD and ALBI Grade) for Disease-Free Survival (DFS) and Overall Survival (OS).

Survival Outcome	Variable	Univariate HR (95% CI)	*p*-Value	Multivariate HR (95% CI)*	*p*-Value
**DFS**	Low PMD (vs. Normal)	1.46 (0.91–2.35)	0.119	1.64 (1.01–2.67)	0.044
	Worsened ALBI (vs. Normal)	3.50 (2.14–5.73)	<0.001	3.00 (1.77–5.08)	<0.001
**OS**	Low PMD (vs. Normal)	2.11 (1.10–4.04)	0.025	1.65 (0.84–3.26)	0.150
	Worsened ALBI (vs. Normal)	2.51 (1.29–4.90)	0.007	2.32 (1.19–4.54)	0.014

*Note: Multivariate models for DFS were adjusted for aggressive tumor factors (pT stage, Fuhrman grade, and LVI), whereas models for OS were adjusted for clinical patient background features (age and CCI).

Table S2. Multivariate Cox Proportional Hazards Regression Analysis for DFS and OS in the Clear Cell RCC Subgroup (n = 244).

Variables	Categories	Multivariate HR (95% CI) for DFS	*p*-Value	Multivariate HR (95% CI) for OS	*p*-Value
**Risk Score**	0	Reference	—	Reference	—
	1	2.17 (1.19–3.95)	0.011	2.72 (1.11–6.67)	0.029
	2	3.91 (1.91–8.00)	<0.001	3.74 (1.30–10.71)	0.014
**pT stage**	≥3 vs. <3	2.37 (1.30–4.30)	0.005	—	—
**Fuhrman grade**	≥3 vs. <3	2.90 (1.66–5.07)	<0.001	—	—
**LVI**	Positive vs. Negative	1.58 (0.85–2.94)	0.149	—	—
**Age, years**	≥63 vs. <63	—	—	2.05 (0.81–5.23)	0.132
**CCI**	≥3 vs. <3	—	—	1.39 (0.62–3.10)	0.425

Note: To maintain consistency with the overall cohort analysis and strictly control for critical confounders, the multivariate model for DFS was adjusted for aggressive tumor factors (pT stage, Fuhrman grade, and LVI), whereas the model for OS was adjusted for patient background features (age and CCI).
